# Measuring Straight Line Segments Using HT Butterflies

**DOI:** 10.1371/journal.pone.0033790

**Published:** 2012-03-27

**Authors:** Shengzhi Du, Chunling Tu, Barend J. van Wyk, Elisha Oketch Ochola, Zengqiang Chen

**Affiliations:** 1 Department of Electrical and Mining Engineering, University of South Africa, Florida, South Africa; 2 F'SATI, Tshwane University of Technology, Pretoria, South Africa; 3 School of Computing, University of South Africa, Pretoria, South Africa; 4 Department of Automation, Nankai University, Tianjin, China; Institute of Psychology, Chinese Academy of Sciences, China

## Abstract

This paper addresses the features of Hough Transform (HT) butterflies suitable for image-based segment detection and measurement. The full segment parameters such as the position, slope, width, length, continuity, and uniformity are related to the features of the HT butterflies. Mathematical analysis and experimental data are presented in order to demonstrate and build the relationship between the measurements of segments and the features of HT butterflies. An effective method is subsequently proposed to employ these relationships in order to discover the parameters of segments. Power line inspection is considered as an application of the proposed method. The application demonstrates that the proposed method is effective for power line inspection, especially for corner detection when they cross poles.

## Introduction

Hough Transform [1] is one of the most widely used and proved effective techniques for locating objects in images [2]–[16]. Detecting straight line segments [11], [14]–[35] in computer vision has received much attention due to the importance of detecting objects/obstacles with straight edges. The HT does not provide a direct method to detect straight line segments, but only the mapping of a segment to butterfly shaped HT data. Using the peak information in HT space is common to all HT related detecting methods. Most segment detecting methods are based on image space feature points verification following the hints of the HT peak. These methods were reported as being highly costly regarding computation [6], [10], [24], [27], [34], [35].

With the exception of the HT peak, micro-analysis of the HT data around the peak has received more and more attention with regards to discovering straight line segment parameters [21], [23], [25], [28], [36]–[38]. In [36], a local operator was proposed to enhance the peak seeking. Furukawa et al. [38] modelled the distribution of butterfly, background, and other objects, and a cross-correlation between the real distribution of a butterfly; the ideal one was used to estimate and evaluate the butterfly. Atiquzzaman et al. [21], [23] reported the micro-analysis of the distribution of the votes around the peak in the accumulator array in order to determine the endpoints of a segment; the length was calculated as the distance between the two endpoints. Kamat et al. [28], [37] discussed the problem of the multiple line segments, where different interesting butterflies were demonstrated due to the different line segments. Du et al. [25] proposed a segment detection method by making use of its quadrangle HT neighborhood, where the position of segments is represented by the position of the center points obtained by detecting the direction of the quadrangle neighborhood. These methods did not verify the feature points in the image space, which renders them computationally efficient.

When considering the fact that all cells in the HT butterfly wings contribute to the representation of the segment, it becomes obvious that detailed segment parameters can be recovered from the information in the butterfly. However, the idea of detecting segments from their butterflies have not received enough consideration in the relevant literature. This paper addresses the features of the HT butterfly and their relationships with the parameters of a segment, and hence demonstrates that the HT butterfly is suitable for segment detection. In this paper, by means of geometric analysis, the authors derive how the full parameters of a segment such as the position, length, width, continuity, and uniformity, are represented by the features of its butterfly. Hence, segments in images can be detected and measured using the formulas derived in this paper. Comparing the various methods [21], [23], [25], [28], [36]–[38], this paper explicitly describes the generation of the butterfly and the relationship between butterfly features and the parameters of segments through simple geometrical analysis. A robust but simple segments discovery method is proposed, where a windowing method is used to remove disturbances from most noise and other objects, and the least square estimations are considered as the reliable segment measurement. Comparing to existing segment detection methods based on HT butterflies, the novelty of the proposed method is manifested in the improvement on the butterflies before detecting segments and the direct relationship between butterfly features and segment parameters which is employed to uncover the full parameters of the segments.

The proposed method is employed in an overhead power line inspection application. Inspection of overhead power lines is an important application aimed at decreasing the time interval of power line disconnections and improves safety during inspection [39]–[41]. The proposed method is applied to the images to detect the overhead power lines. Specifications for this application were used to distinguish disturbing objects from power lines. The detection results indicate that the proposed method is effective.

## Methods

### HT and HT butterflies

In HT, each feature point 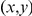 of a segment is mapped to a sine curve via the following equation:
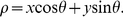
(1)


By discretizing the HT space by resolutions of 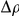 and 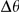, the feature point 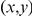 votes for every cell located on the curve. After the voting process, the position of the cell receiving the most votes, that is, the peak, is considered as the 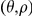 values of the segment. Obviously, the HT converts the pattern recognition problem to a peak generating and seeking problem. This makes the HT robust to the noise and complex background. How to generate a strong and distinct peak and how to seek the “correct” peaks is one of the most focused research on HT where the computational complexity, storage requirement, accuracy, and resolution are widely considered, especially the situations of high accuracy and high resolution HT is required. Various methods were proposed to improve the voting process [2], [12], [16], the discretization of the HT space [11], feasible resolutions settings [13], post-voting process [3], [6], [19], [27], [34], etc. These research works put most emphases on the distinct peaks generating and seeking, and hence can be called “peak based” methods. A common problem of these methods is the infeasible computational complexity and memory storage requirement [5], [7], [29], [32], [33].

**Figure 1:**
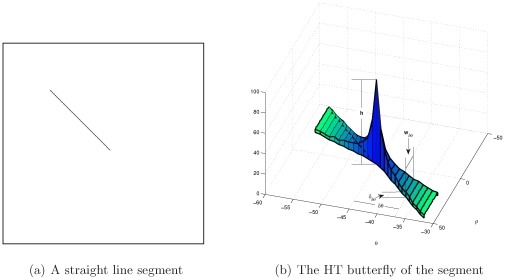
A straight line segment and its HT butterfly.

Another important branch of HT researches is the micro-analysis on the “HT butterfly”. In fact, during the voting process, not only the peak is generated, but also, a large area composed of all mapped sine curves is built. By considering the part of this area around the peak, a butterfly shape is obtained. For example, the segment displayed in Fig. 1(a) is mapped to the HT space and considering only the area around the peak, the butterfly-shaped voting area shown in Fig. 1(b) can be obtained where the height of the peak is denoted as 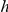, the thickness of the wings at 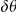 far from the peak is denoted as 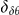, and the width of the wings is 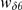.

**Figure 2:**
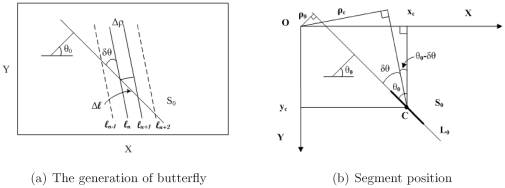
Principle of geometric derivation.

Fig. 2(a) demonstrates how the butterfly is generated. For a given segment 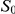 lying on a straight line 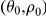, the HT cells lying on the column with distance 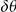 to the HT peak correspond to the belts bounded by parallel straight lines 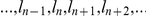. The angle between 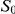 and these straight lines is 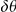. All the belts that intersect with 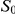 will contain some feature points of 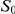 and hence the corresponding cells will receive some votes. With 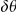 increasing, the number of belts intersecting with 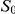 increases and the feature points contained in each belt decreases. This means that when the column moves further from the peak, more cells will receive votes. Therefore, a butterfly shaped voting area is generated with 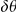 increasing from 0 to a given value. In this manner, given a segment and the scope in HT space, a *unique* butterfly can be generated. It implies that if the butterfly is known, one can expect to cover the segment from its HT butterfly. This section shows the relationships between the parameters of the segment and the features of its butterfly. Each parameter of the segment can be represented by one or more features of its butterfly.

The “HT butterfly based” methods use not only the peak but also the data in the area around the peak [20]–[23], [25], [28], [36]–[38], [42]–[44]. Because of the 1–1 mapping between a segment and its HT butterfly, these “HT butterfly based” methods have potentials to uncover high accuracy segment parameters from low resolution HT data. The features of the butterfly are popularly discussed and employed in these methods, such as the self-similarity [42] and the symmetry [43] are used to improve the resolution and accuracy of HT. The features of butterflies are used to identify and enhance the peak [36], [38].

Collinear segments detection is another important extension of the “HT butterfly” to the commonly used “peak based” HT methods. Because the butterflies of collinear segments intersect on a common peak and are independent/separated at the area beyond the peak, the “peak based” methods obviously lose the distinguishing ability of these segments. However, the “HT butterfly based” methods have this distinguishing ability naturally [25], [44].

### Representation of segment measurements by the features of its HT butterfly

#### Segment length vs the width of its HT butterfly wings

The generation of the butterfly demonstrated in Fig. 2(a) clear indicates that for a given 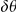 a longer segment leads to more of the cells in the column receiving votes. This implies that the butterfly wings of a longer segment is wider than the ones of a shorter segment. Therefore, it is possible to detect the segment length by means of the width of its butterfly wings.

In Fig. 2(a), it is obvious that the length of the intersection of the segment and a belt (if they intersect) can be obtained as follows:
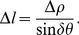
(2)


Therefore, the number of cells in the column, that is, the width of the butterfly wings, corresponding to 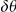 can be used to obtain the length of the segment as follows:
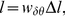
(3)where 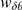 is the number of cells receiving votes in the column of 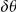 as shown in Fig. 1(b), 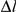 is the length of the intersection of the segment and the belt shown in eq. (2), and 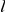 is the detected length of the segment.

It should be noted that the belts that intersect with the segment at both ends might contain fewer feature points than the ones in the middle, implying that the length of these intersections are smaller than the 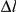 shown in eq. (2). Hence the detected length may be bigger than the ‘true’ value by up to 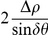. This error is depressed when the 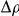 is small enough and 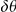 is large enough.

#### Segment width vs the thickness of its HT butterfly wings

The demonstration in Fig. 2(a) indicates that for a given 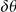 the length of the intersection between the segment and the belt is fixed and can be obtained, likewise the number of feature points contained in the intersection, that is, the number of votes received by the corresponding cell, is related to the width of the segment. It is obvious that each intersection, except the ones at both ends, contains similar number of feature points if the segment is uniform. Considering that the number of votes received by the cell is represented by the thickness of the butterfly wings, the thickness of wings is nearly constant for a given column. Therefore, it is possible to derive the segment width via the thickness of its butterfly wings.

Considering the nature of the digital images used in the computer, all straight lines are represented by piece-wise connected horizontal or vertical short segments. The number of pixels composing a straight line is equal to the projection of the straight line on 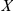 (when 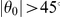) or 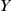 (when 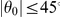) axes. For a single-pixel width line, the number of pixels contained in the belt corresponding to 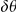 as shown in Fig. 2(a), that is, the thickness of the butterfly wings corresponding to 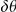, is:
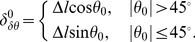
(4)Substituting eq. (2) to eq. (4), one obtains
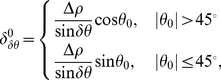
(5)where 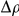 and 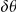 are predefined, and 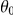 can be obtained from the butterfly, hence 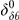 can be determined.

Given the butterfly and the value of 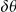, the width of the segment can be obtained as follows:
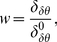
(6)where 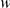 is the width (counted by pixel) of the segment, 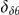 is the measured thickness of butterfly wings, and 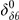 is obtained by eq. (5).

#### Segment position vs the direction of its HT butterfly wings

One can obtain the 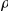 and 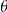 values of the straight line to which a segment belongs. This section solves the problem of “where is the segment on the straight line?”

The position of the segment, that is, its center point position, relating to the direction of its butterfly, was approved in [25]. This can also be explained by Fig. 2(a), where the position of the segment determines the 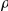 values of the belts intersecting with it, that is, the position of the HT cells in the column corresponding to 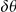.

Fig. 2(b) shows a segment 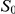 lying on the straight line 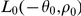. The center point of 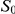 is 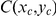. When the cells on the column corresponding to 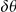 are considered, the cell lying on the center of the butterfly wing corresponds to the belt containing center point 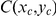 of the segment. From Fig. 2(b) one obtains:
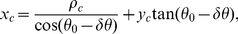
(7)where 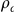 is the 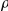 coordinate of the cell lying on the center of the column in the butterfly wing corresponding to 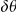. Considering 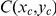 lying on the straight line 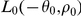, one obtains:
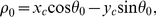
(8)i.e.
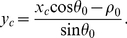
(9)By substituting eq. (9) into eq. (7) one obtains:
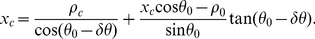
(10)By solving eq. (10), one obtains:
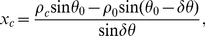
(11)where 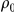 and 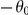 can be detected by seeking peaks in HT space, 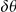 is predefined, and 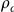 can be measured in the column of HT space corresponding to 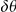.

One can obtain 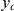 by substituting eq. (11) into eq. (9).

#### Segment continuity and uniformity vs the non-uniformity of its HT butterfly wings

In practical applications, collinear segments are popular and sometimes straight lines are not uniform, that is, the width of different parts of the line is different. This section demonstrates how the following information is represented in HT butterflies: “How many collinear segments are contained in a straight line?”, “What are their parameters?”, “Is the segment non-uniform” and “How non-uniform is a segment?”

A straight line containing several collinear segments means gaps exist between these segments. Fig. 2(a), indicates that appropriate 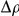 and 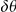 causing one or more belt to intersect with the straight line in a gap. These belts contain very few or no feature points and hence the corresponding cells receive very few, if any, or no votes. This means that the butterfly of the segment containing all collinear segments is gapped to several smaller butterflies. It is obvious that the number of collinear segments can be obtained by counting the number of small butterflies sharing the same peak. The parameters of each segment can be detected using the methods mentioned in this paper.

A non-uniform segment can be considered to be composed of continuous segment(s) and some adjacent collinear short segments. The butterflies of these segments will be superposed in the HT space. Therefore, the non-uniformity of HT butterfly wings implies the non-uniformity of segments in the image space.

### Isolating the Butterfly of a Single Segment for Measurement

The above section demonstrated how a butterfly is generated from a segment and how the segment is measured via the butterfly. This section focuses on how to achieve the butterfly of a single segment from the HT data of a given image, so that the segment parameters can be obtained by the formulas derived in this paper.

Of course the peak is the geometric center of a butterfly, which can be detected by seeking the maximum in the HT data. By denoting the peak position as 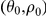, the column having 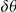 distance to the peak (i.e. the column 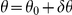 or 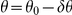) corresponds to the set of belts having 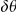 angle with the segment as shown in Fig. 2(a). Each belt intersecting with the segment contains part of the feature points of the segment, implying that the cell corresponding to the belt gets votes from the segment. The number of votes gotten by the cell depends on the length of the intersection, i.e. the 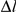 in eq. (2). It should be noted that from eq. (5) one finds for all cells in a column of the wings get the same number of votes in theory.

After seeking the peak from the HT data of an image, it is easy to isolate the butterfly around the peak by ignoring all cells getting votes less than the theoretical thickness of the column they are associating with. In fact, considering the discretization error of an image and HT space, and rounding error of the voting process, the threshold should be a bit lower than the theoretical thickness.

To eliminate the disturbances from other peaks, the windowing method proposed in [44] is employed, i.e. a neighborhood of the segment in the image space is mapped to a sub-HT space.

### A Robust Method of Discovering Segment from Butterflies

**Figure 3:**
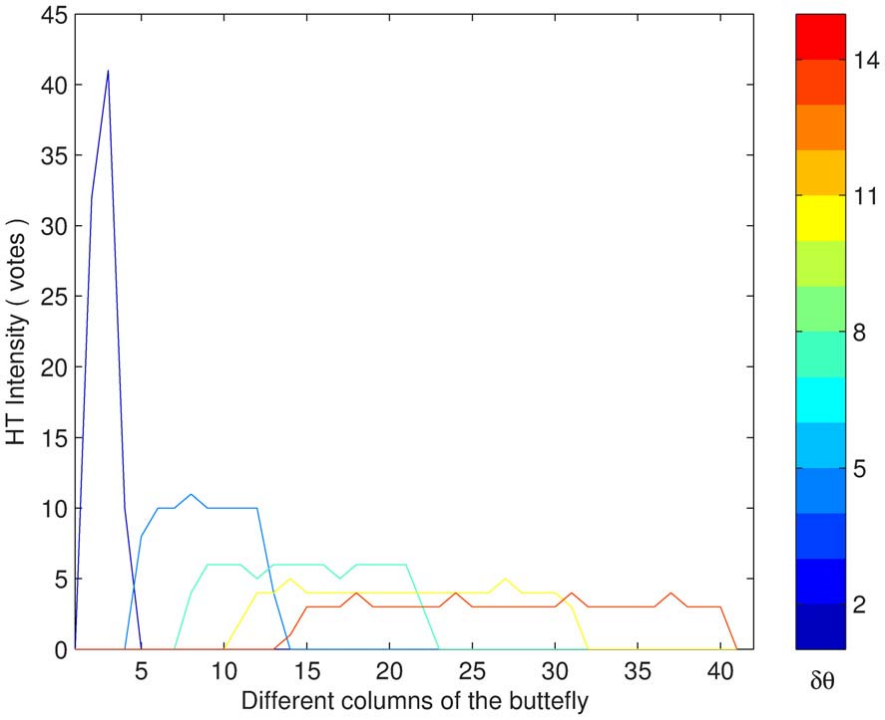
Columns of segment butterfly.

In above sections, the segment parameters can be discovered in a single column of the butterfly. However, due to the existence of rounding errors during the digital imaging, voting and discretization, each column in the HT butterfly might display different parameter values. For example, the image in Fig. 1(a) has the different columns of its butterfly displayed in Fig. 3.

**Figure 4:**
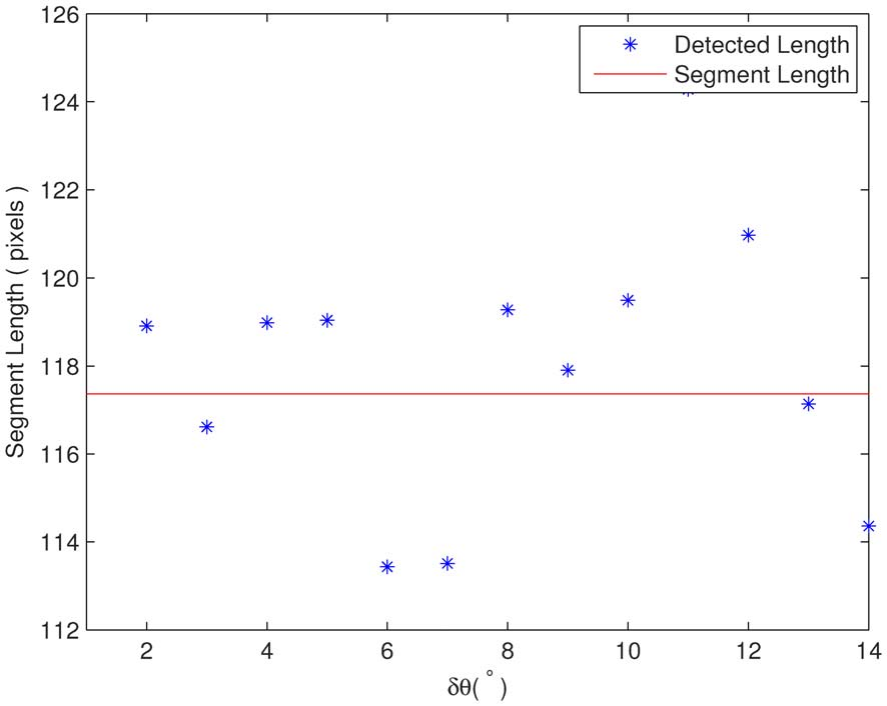
Detected segment length.

The corresponding width, intensity, and center point of these columns are effected by these errors. The detected parameters are also effected; for example, the segment lengths detected by different columns as shown in Fig. 4 are unreliable; where the detected length is distributed around the “true” value. For the sake of robustness, the Mean Square Error (MSE) estimations are considered as the reliable parameter values obtained from the observations of the different columns.

**Figure 5:**
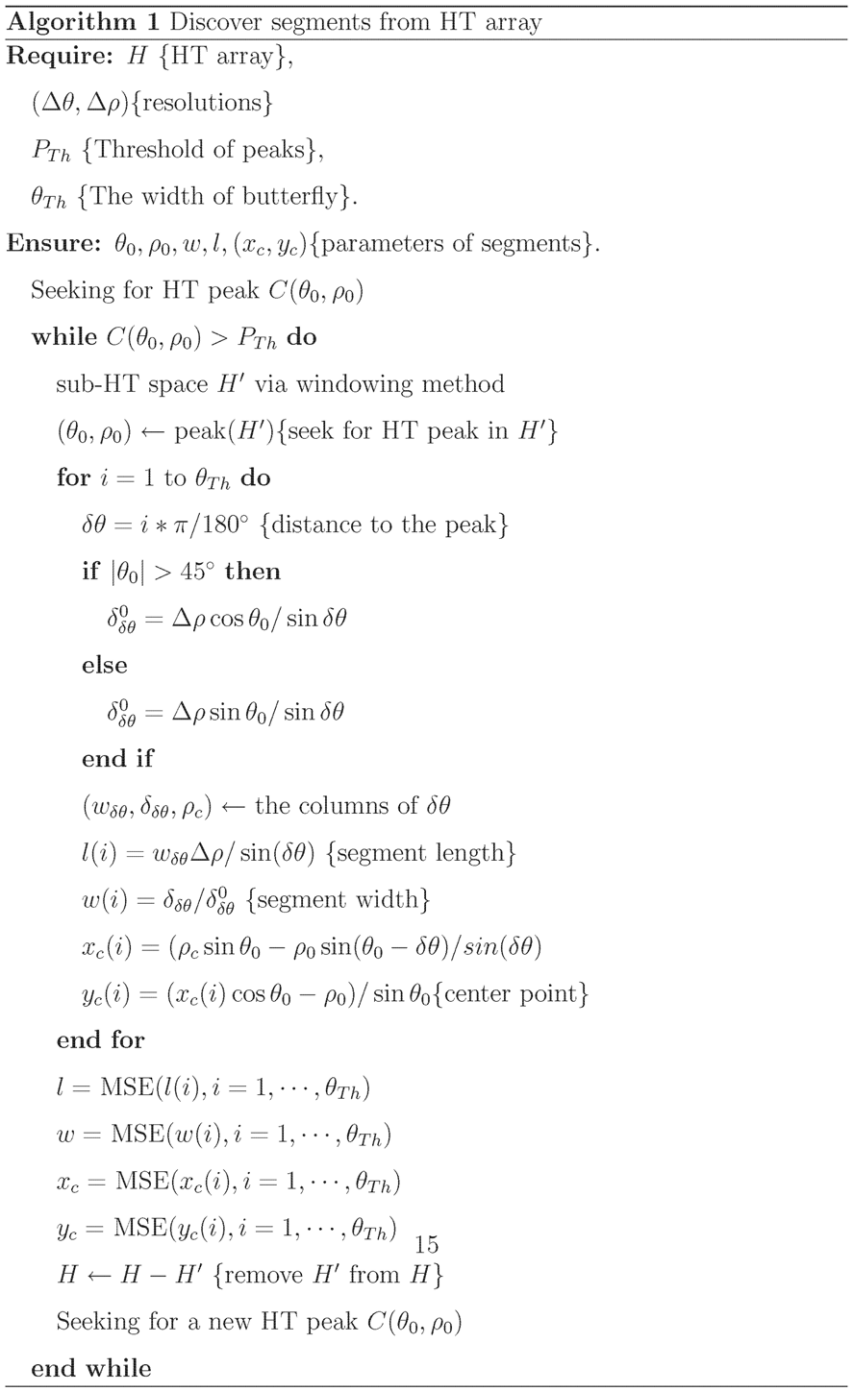
Discovering segments from HT array.

The algorithm (Fig. 5) pseudocodes summarizes the proposed method.

### Specifications for Power Line Inspection

This paper proposed a general method for segment detection via its HT butterfly. When the proposed method is employed in specified applications such as power line inspection, usually this is existing heuristic information present that might reduce the associated complexity, uncertainties, and difficulties. By reasonably utilizing this information benefits of obtaining simple and reliable solutions emerge as follows:

1. Length: since the objects to be detected are power lines and the camera is supposed to be facing to the objects, the objects (segments) length should be considerable. That is, the disturbing segments shorter than a predefined length could be ignored;

2. Direction: the power lines are usually parallel and hence the image usually contains several parallel segments, so the solitary segments running in obviously different direction compared to others could be ignored;

3. Width: for the sake of safety, the images were taken with a considerable distance to the power lines and the power lines are usually not too thick (with diameter up to several centimeters), hence the objects with obviously unreasonable width (such as the poles and the insulators) could be ignored;

4. Number: at least two power lines are needed in power delivery, so the number of segments running in similar directions should not be less than 2;

5. Distance: the minimum distance between any two power lines is strictly specified, hence the parallel segments having very small distance should be ignored;

6. In-out logic: when power lines run crossing poles, especially in the case of designed turn after the pole, the number of power lines “getting in” the pole should be equal to the number of “getting out” ones, and an intersection can be expected on the extension of a pair of “in” and “out” power lines.

## Results

### Experiments

#### Segment Length vs Butterfly Width

**Figure 6:**
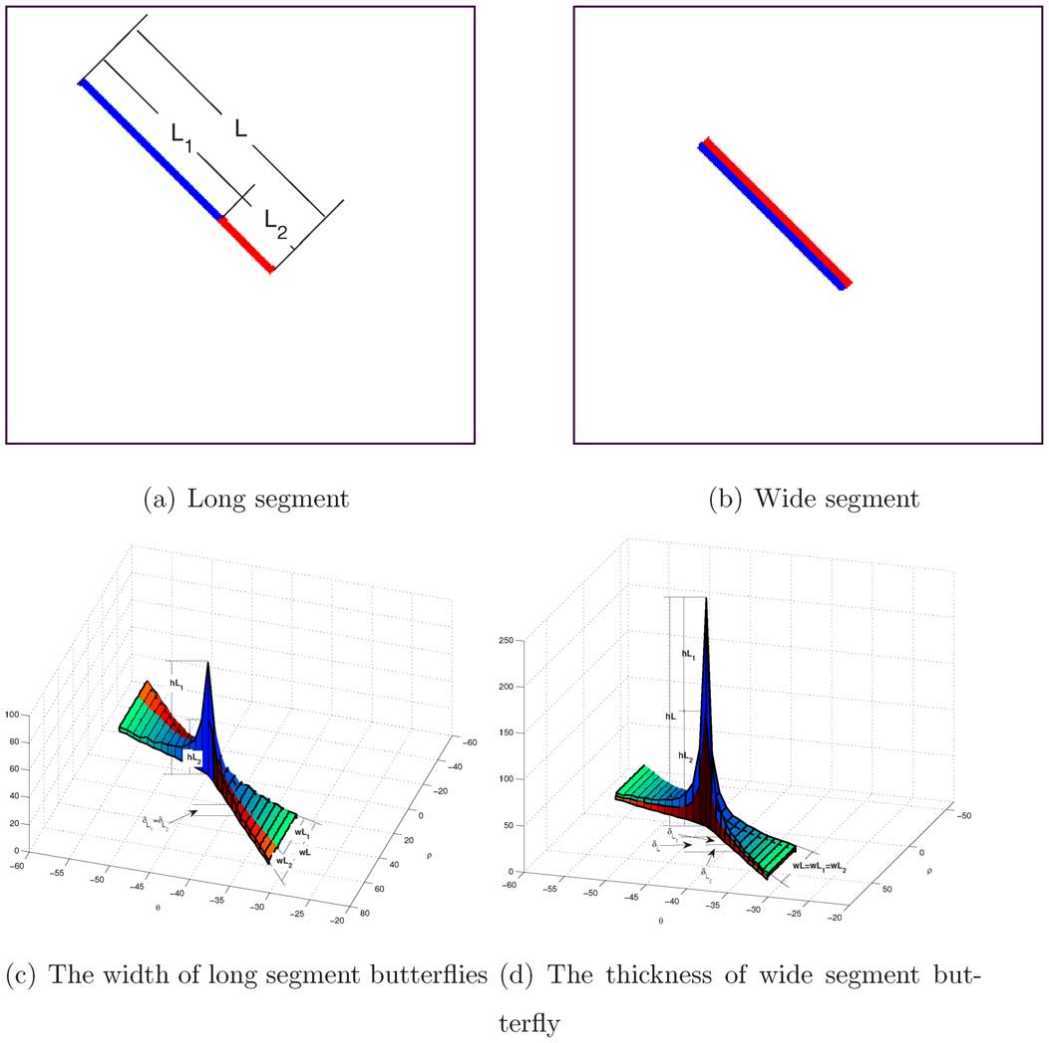
Segment length and width.

Fig. 6(a) depicts a segment composed of two connected parts. Fig. 6(c) shows the HT data of the segment, where each part corresponds to a butterfly, and the two butterflies perfectly merge into a bigger butterfly. This occurs because the two parts are connected without any gap. The width of the merged butterfly is equal to the sum of the width of the two small butterflies, that is, the length of the segment is equal to the sum of the length of its two parts. This experiment verifies that detecting the segment length via the width of its butterfly wings is justifiable.

#### Segment Width vs Butterfly Thickness

Fig. 6(b) depicts two adjacent segments and Fig. 6(d) portrays their HT butterflies. In the image space, the two segments merge into a wider segment with the same length. In the HT space, the butterfly of the merged wider segment comprises only the superposition of the butterflies of the two narrow segments, because the width of the merged segment is equal to the sum of the width of the two narrow segments, and hence the thickness of the butterfly of the former is equal to the sum of the thickness of the butterflies of the latter. This verifies the linear direct proportion between the width of a segment and the thickness of its butterfly wings.

#### Segment Position vs Butterfly Direction

**Figure 7:**
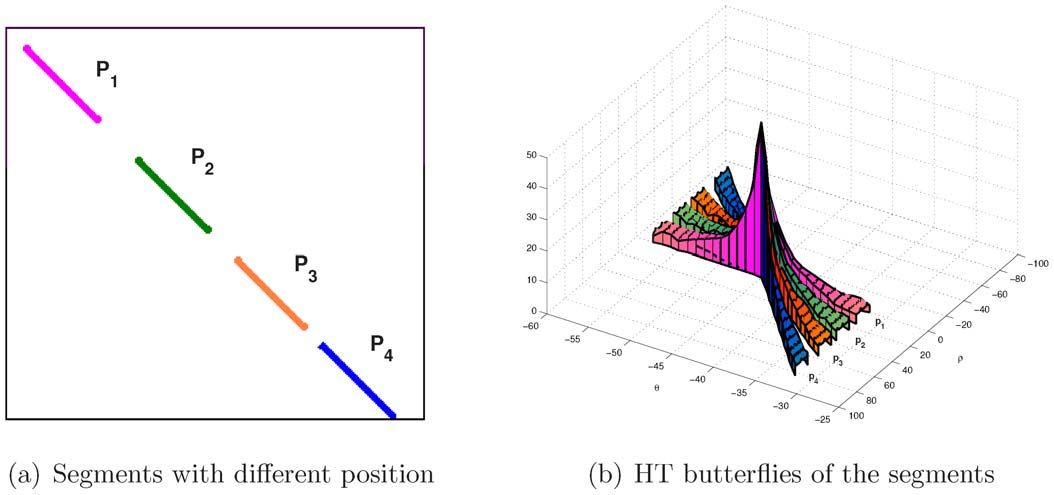
Segment position vs its HT butterfly direction.

Fig. 7(a) depicts a segment as it changes its position on the straight line to which it belongs, and Fig. 7(b) indicates the butterflies corresponding to the different positions of the segment.

#### Segment continuity and uniformity vs the butterfly non-uniformity

**Figure 8:**
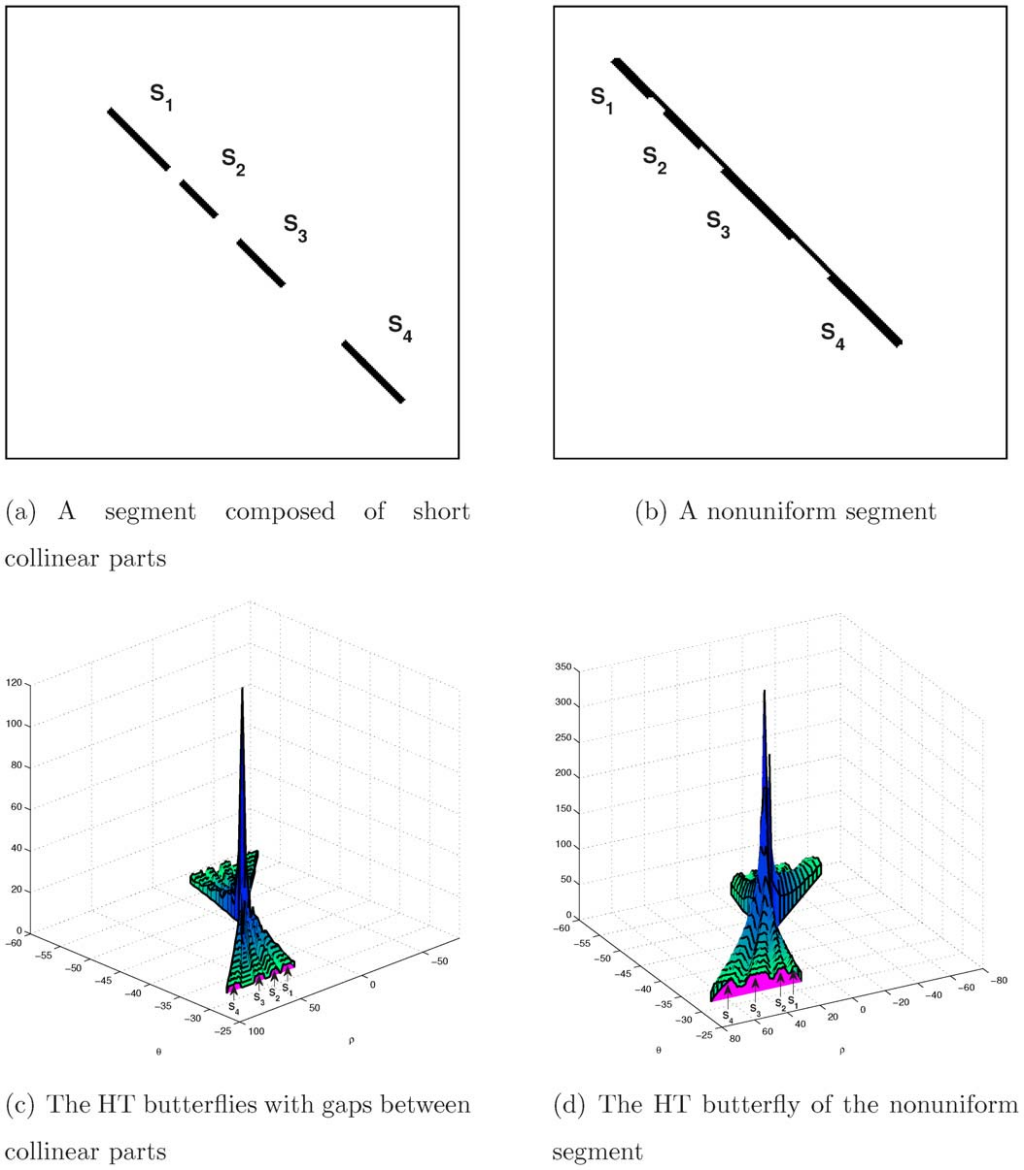
Uniformity of segment and its HT butterfly.

Collinear segments 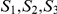 and 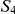 are illustrated in Fig. 8(a). The HT data is shown in Fig. 8(c), where the butterflies of these segments are gapped by valleys (the sections that receive no votes). Because all these segments lie on the same straight line, their peaks are located at the same position in the HT space.

Fig. 8(b) demonstrates the case of a non-uniform segment. Fig. 8(d) shows the HT data of the segment in Fig. 8(b), where the wings of the butterfly are non-uniform.

### Comparison with Existing Butterfly Region Based Segment Detectors

Experiments in this section aim to compare the performance of the proposed method with those also based on butterfly region proposed in literatures, such as the method of Atiquzzaman [21], [23] (denoted as Method I), and the method of Kamat [28] (denoted as Method II). The cases of thick segments, collinear disturbances and collinear segments are considered for comparison.

#### The effect of segment width on detection performance

**Figure 9:**
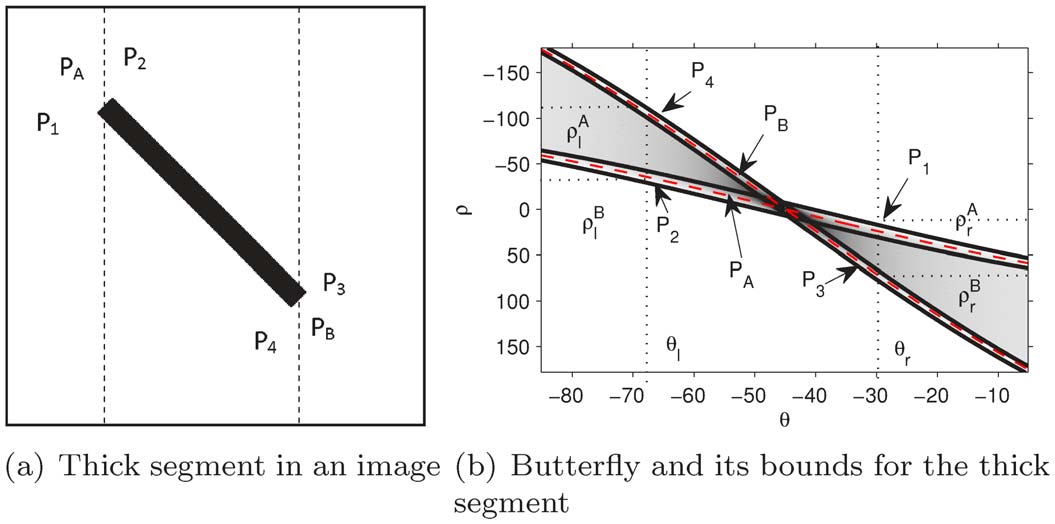
Comparison of thick segment detection.

Methods I and II analyze one or more columns of accumulator cells around the peaks to find the first and the last non-zero cells, and then calculate the end points of segments. The width of segments affect the detection accuracy. Fig. 9(a) shows a thick segment in image space (the points 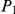, 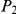, 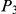, and 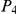 are its vertexes, 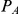 and 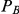 are its end points) and Fig. 9(b) shows its butterfly region around the peak. From the HT data, it is observed that the first non-zero cell of the columns on the left side of the peak corresponds to the vertex 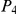, and the last non-zero cell corresponds to the vertex 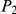. The first and the last non-zero cells of the columns on the right side of the peak correspond to vertices 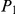 and 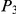, respectively. In fact, for the case of thick segments, Methods I and II only detect the diagonals (i.e. 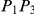 and 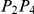) instead of the segments. The proposed method solves this problem by detecting segments using the central line of the butterfly. Obviously, the central line of the butterfly is not affected by the segment width. The proposed method outperforms Methods I and II in terms of reliability and accuracy when detecting thick segments.

#### The effects of disturbances/noise on detection performance

**Figure 10:**
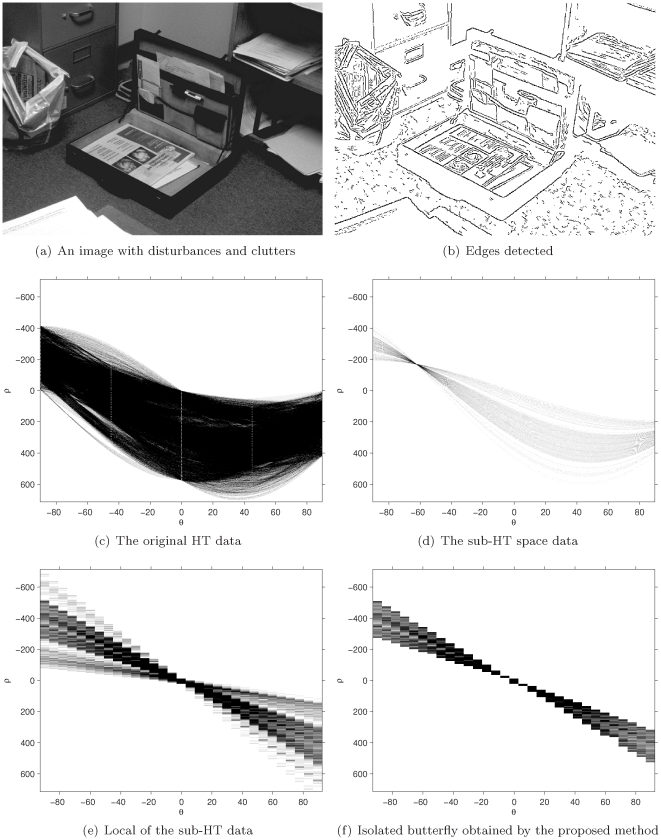
The butterfly improvement obtained by the proposed method under the situation of the disturbances and clusters in a real image.

This experiment addresses the effects of disturbances/clutters on the segment detection. Fig. 10(a) is an image having clutters and noise. Fig. 10(b) is the result of edge detection. Fig. 10(c) is the HT data of Fig. 10(b) where the butterfly is badly degraded due to the existences of other objects, clutters and noise. Obviously, methods I and II cannot correctly detect the first and the last non-zero cells for a given column of the accumulators in Fig. 10(c). No appropriate window described in Method II exists to isolate the butterfly from clutter because the peak lies on the area overlapped by the HT mapping of quite a number of other objects and disturbances/clutters.

Fig. 10(d) is the HT data after the sub-HT space method is employed. Fig. 10(e) shows the part around the peak in Fig. 10(d), where the butterfly becomes quite clear because most disturbances/clutters are removed but only collinear ones left. Although the butterfly in this sub-HT space is quite improved comparing with the one in Fig. 10(c), Method I and II still cannot correctly detect the segment because the butterfly is still degraded due to the existences of disturbances/clutters lying collinearly with the object, which means Method I and II are very sensitive to collinear disturbances.

Further improvement is obtained (as shown in Fig. 10(f)) by employing the butterfly isolating method proposed in this paper, where the edge of the butterfly becomes very clear and the effects of most collinear disturbances/clutters are removed. This means the proposed method has immunity from collinear disturbances and hence outperforms Method I and II. Of course, based on this improved butterfly, not only the proposed method but also all the methods based on butterflies can get a much better detection performance.

### Application on Real Image

**Figure 11:**
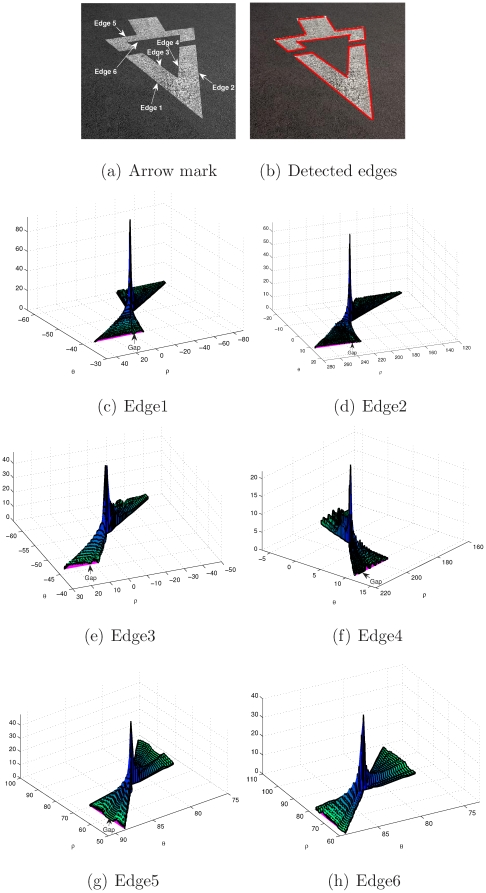
Butterflies for each edge.

Fig. 11(a) displays an arrow with numbered edges. Fig. 11(b) indicates the arrow's detected edges. Fig. 11(c)–(h) show the butterfly of each edge. In Fig. 11(a), it is obvious that the first 5 edges (i.e., from Edge 1 to Edge 5) are composed of two collinear segments. Fig. 11(c)–(g) clearly reveal the gap between these collinear segments, which is the proof for considering that these edges are discontinuous.

### Power Lines Inspection

**Figure 12:**
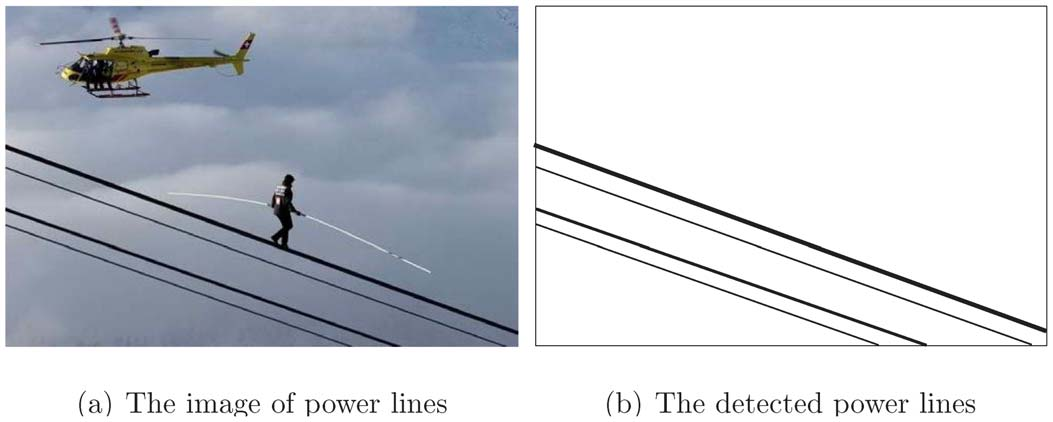
The case of power lines crossing the view field.

Several images containing power lines are used to verify the proposed method. Fig. 12 shows the case of power lines running across the whole view of the camera, where the power lines that do not have turns in the image are shown in Fig. 12(a). The corresponding detected power lines are displayed in Fig. 12(b). All other disturbing objects are excluded by applying the specifications mentioned in this paper.

**Figure 13:**
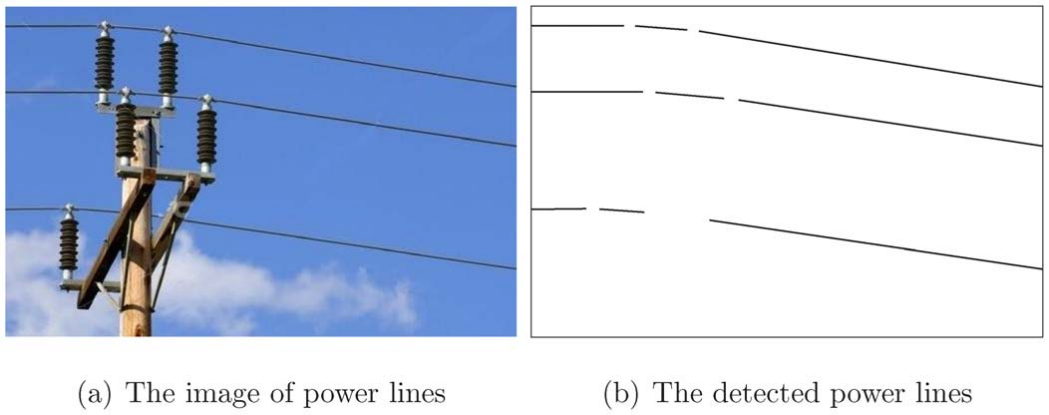
The case of power lines turning due to the gravity.

Fig. 13(a) demonstrates the case of power lines turning downwards due to the gravitation force when they cross the pole. Fig. 13(b) displays the image of the corresponding detected power lines. As disturbing objects, the pole and the insulators on the top of the pole are excluded due to the width specification.

**Figure 14:**
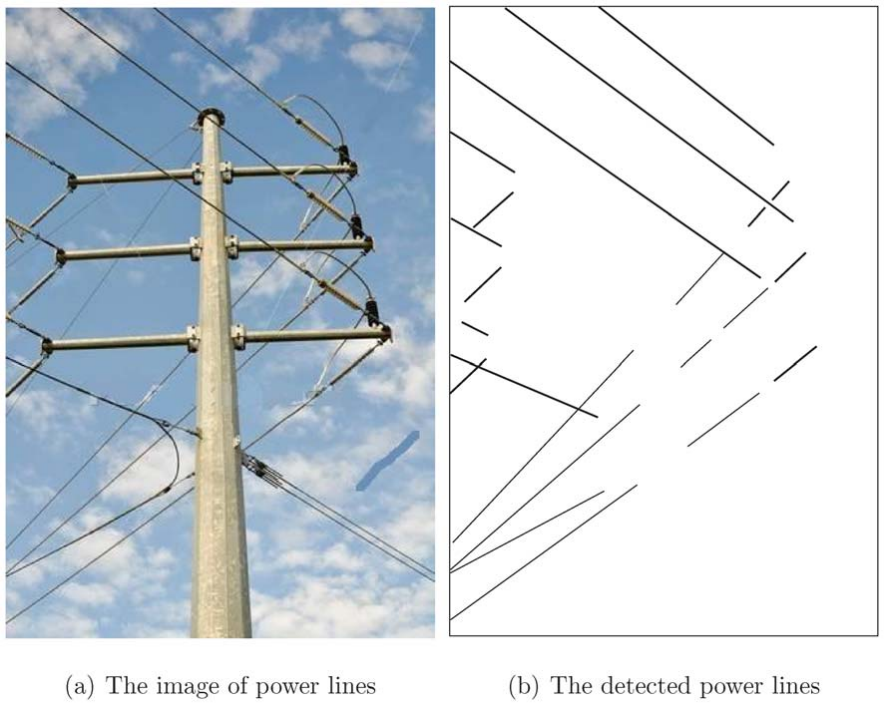
The case of power lines turning due to the designed turn.

Fig. 14 shows the case of designed turns when power lines run across a pole. The pole and insulators are similarly excluded due to the width specification and the lifting ropes are excluded owing to the direction, “in-out” logic, and distance specifications.

## Discussion

In this paper, the authors formulated the segment measurement by means of simple geometric analysis. The measurement of full parameters of a segment is discussed and represented by the features of its butterfly, including the length, width, position, continuity and non-uniformity of the segment. The relationship between butterfly features and segment parameters are demonstrated in this paper, and the illustrations show that it is reasonable to detect and measure segments via their butterflies. The involvement of HT data around peaks renders the relationship independent of the sharpness of the peaks. Based on these relationships, an effective and robust segment measuring method is proposed and applied to synthetic and real images in order to demonstrate the performance. The experiments verify the proposed method.
